# 606 Novel application of a surgeon-operated clysis delivery system in burn surgery

**DOI:** 10.1093/jbcr/irac012.234

**Published:** 2022-03-23

**Authors:** Alexander Morzycki, Joshua N Wong

**Affiliations:** Division of Plastic and Reconstructive Surgery, University of Alberta, Edmonton, Canada, Edmonton, Alberta; Division of Plastic Surgery, Department of Surgery, University of Alberta, Edmonton, Alberta

## Abstract

**Introduction:**

Tangential excision of burns is associated with significant bleeding. Sub-eschar insufflation of epinephrine-containing clysis has shown to decrease blood loss and associated complications. Administration of adrenaline-containing infiltrates are also beneficial in the harvest of split thickness skin grafts. Clysis is typically delivered with the assistance of a perfusionist-operated system. This method, however, is associated with significant cost and dependent on personnel availability. This study evaluated the use of a novel surgeon-operated fluid management system in the delivery of clysis in burn surgery.

**Methods:**

Our initial experience with a novel fluid management system is presented. Prospective collection of infiltration data, including average temperature, pressure, and volume of clysis was performed. Patient and burn factors were evaluated and complications collected. Finally, a cost-effectiveness analysis was conducted.

**Results:**

Thirty-seven consecutive cases comprising 22 adult patients (15/22, 68% male), with a mean age of 49 years (+/- 19) were reviewed. The mean % total body surface area of all patients was 39 (+/- 21.7). The mean temperature, pressure and volume of administered clysis was 32.2 degrees Celsius (+/- 4.4), 265.04 mmHg (+/-56.17), and 5805.8 mL (+/- 4844.4), respectively. The mean dose of epinephrine administered was 14.5mg (+/- 12.1). The mean temperature variability was 1.1 °C (+/- 1.2). Total mean packed red blood cells (PRBC) transfused was 507.6 mL (+/- 624.4). There were no recorded complications. We identified a cost savings of $20,766 CAD over the cases examined.

**Conclusions:**

We present the novel application of a fluid management system in burn surgery. This technique provides rapid and safe infiltration of warmed clysis. We are able to maintain intra-operative euthermia despite a large volume of administered clysis and significant intraoperative vulnerability to hypothermia. In addition, this technique may be transfusion-sparing. The introduction of this method of clysis administration was associated with significant cost-savings.

